# Leveraging Language Model Multitasking To Predict C–H Borylation Selectivity

**DOI:** 10.1021/acs.jcim.4c00137

**Published:** 2024-05-06

**Authors:** Ruslan Kotlyarov, Konstantinos Papachristos, Geoffrey P. F. Wood, Jonathan M. Goodman

**Affiliations:** † Yusuf Hamied Department of Chemistry, University of Cambridge, Lensfield Road, Cambridge CB2 1EW, U.K.; ‡ Exscientia Plc, The Schrödinger Building, Oxford Science Park, Oxford OX4 4GE, U.K.

## Abstract

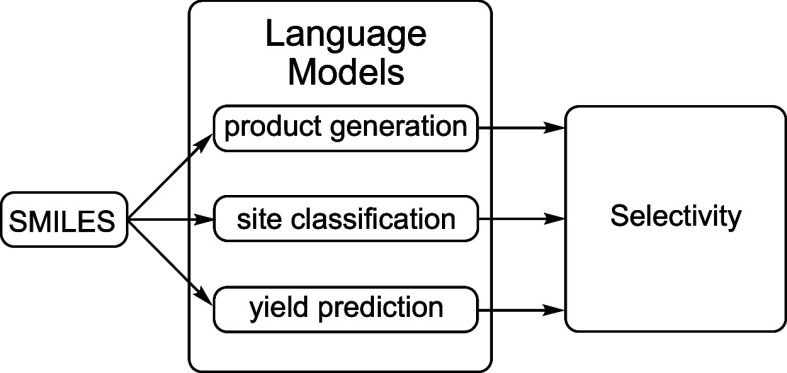

C–H borylation is a high-value transformation in the synthesis of lead candidates for the pharmaceutical industry because a wide array of downstream coupling reactions is available. However, predicting its regioselectivity, especially in drug-like molecules that may contain multiple heterocycles, is not a trivial task. Using a data set of borylation reactions from Reaxys, we explored how a language model originally trained on USPTO_500_MT, a broad-scope set of patent data, can be used to predict the C–H borylation reaction product in different modes: product generation and site reactivity classification. Our fine-tuned T5Chem multitask language model can generate the correct product in 79% of cases. It can also classify the reactive aromatic C–H bonds with 95% accuracy and 88% positive predictive value, exceeding purpose-developed graph-based neural networks.

## Introduction

Late-stage functionalization (LSF) of C–H bonds is an important approach to lead compound development in the pharmaceutical industry.^1−3^ LSF can be used for both fine-tuning the structure of a lead and supporting extensive structure–activity relationship studies. The preference for C–H bonds is both an advantage due to their ubiquity and a drawback due to the difficulty of differentiating similar bonds. While for simple cases it is possible to derive a set of heuristics for site selectivity, the presence of multiple competing factors necessitates more complex models.

Iridium-catalyzed C–H borylation is an example of such reaction. Its products are safe to handle and can undergo a wide range of cross-coupling reactions, forming C–C bonds via Suzuki-Miyaura reaction^4,5^ or connecting to heteroatoms via Chan-Lam-Evans coupling.^6−8^ This makes organoboronates ideal candidates for streamlined modular drug candidate synthesis.

**Figure 1:**
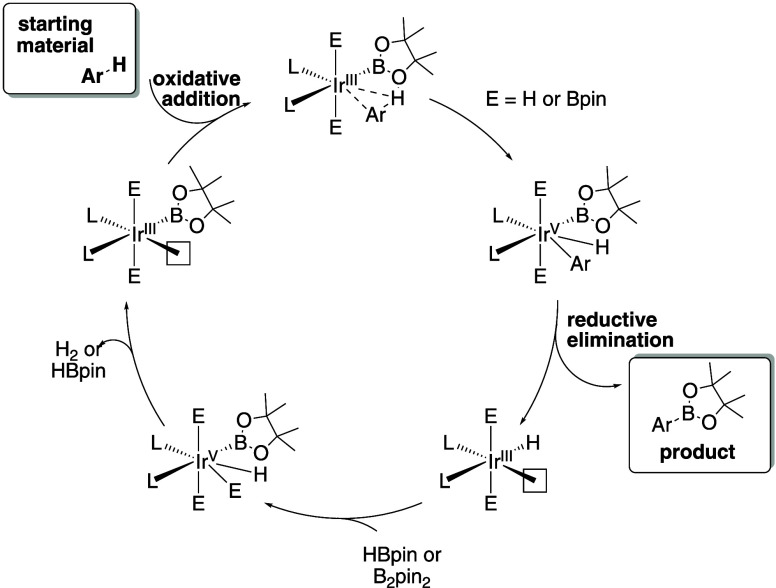
Mechanism of undirected iridium-catalyzed borylation (resting state omitted).

The mechanism of the reaction has been determined (Figure 1) and can be used to explain the selectivity.^9^ For aromatic compounds, the rate-determining step is an irreversible oxidative addition^10^ to the C–H bond in a substrate, and the reaction proceeds faster at sterically unencumbered acidic C–H bonds. Studies of heterocyclic substrates^11^ helped to derive a set of guidelines for borylation selectivity: the reaction avoids taking place next to ortho substituents or basic nitrogen atoms, electron-deficient heteroarenes react faster than arenes, and 5-membered heterocycles are preferred to 6-membered heterocycles, probably due to steric factors.

The complexity of the competition between electronic and steric factors rapidly increases with the size of the molecule. For example, if there are multiple aromatic rings, which one would react and which bond of this ring would be preferred? For complex systems, simple heuristics are insufficient, and a different approach is needed.

## Prior Art

### Quantum Mechanics-Based Models

Ab initio quantum-mechanics modeling has been used in elucidation of the catalytic cycle of C–H borylation.^10,11^ Calculating the reaction barriers for each position is an effective way to determine the selectivity, including the stereoselectivity, but it is also computationally expensive because of the variety of catalytic pathways, active species, and solvent effects coupled with the effort required to find each transition state.^12^ Performing such calculations for every possible reaction site may end up being uncompetitive to running an experiment. It is, therefore, necessary to consider approximations, simplifications, and alternative methods to speed up selectivity prediction.

**Figure 2:**
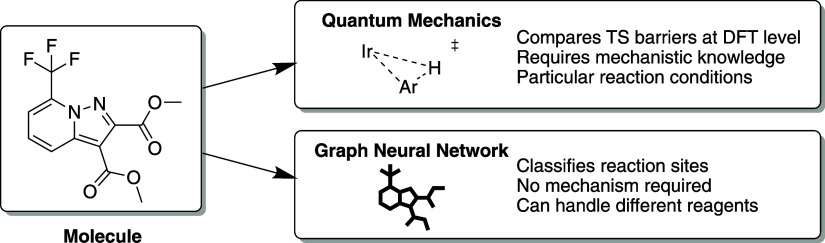
Overview of existing approaches for regioselectivity prediction.

Noting that the selectivity for iridium-catalyzed borylation is controlled at the oxidative addition step (Figure 1), researchers from AstraZeneca and UC Berkeley have built the hybrid SoBo model^13^ to predict relative barriers for each position (Figure 2, top right). The model uses a transition state for benzene preoptimized at the density functional theory (DFT) level and uses a semiempirical quantum mechanical method to get the approximate barrier heights. The predictions are refined further using a combination of two correction terms. The first term is the neighbor penalty, which estimates steric bulk in the *ortho* position next to the reaction site. The second term is a partial least-squares regressor that models the local chemical environment. Depending on the similarity of the training set, these two correction terms are combined dynamically. Using the SoBo model, the authors found that the prediction can be generated in minutes using a desktop computer as opposed to hours on a high-performance cluster for a traditional DFT calculation. The model does not take the absolute barrier height into account, which means it cannot predict whether the reaction is fast or slow.

### Graph Neural Networks

The connectivity of a molecule can be represented as a 2D graph, and additional features such as bond lengths can be incorporated into 3D graphs. Within the network, the atoms and their connections are associated with a set of features, including atom types, ring membership, aromaticity, and atom hybridization, which are commonly known as embedding vectors. These features can be updated with features of neighboring atoms or connections. After several iterations, each expanding the number of atoms that influence each feature, the model should be able to take long-range interactions between the atoms into account. A graph-based approach for C–H borylation has been developed by researchers from Roche, LMU, and ETH^14^ (Figure 2, bottom right). To model regioselectivity, an atomistic graph neural network (aGNN) architecture was employed that represents the borylation substrate as a molecular graph.

This model was applied to a selectivity task: Which nonquaternary carbon atoms are reactive? While the lowest accuracy model, aGNN2D, which used only 2D information, gave 88% accuracy, the *F*-score was only 38% with true positive at 30%, demonstrating how the class imbalance distorts the metrics. The use of 3D structures to initialize the graphs (aGNN3D) improved the accuracy to 90%, and the true positive rate improved to 56%, demonstrating that the model was now much more effective. Augmenting the graphs with DFT-accuracy,^15^ Mulliken partial charges for each atom (aGNN2DQM, aGNN3DQM) had a negligible impact on the metrics.

The approach was restricted to carbon (C), hydrogen (H), oxygen (O), nitrogen (N), sulfur (S), phosphorus (P), and the halogens. The site-level accuracy metrics (whether the reactivity of a C–H bond is predicted correctly) do not reflect the accuracy of an overall molecular-level prediction: What is the major reaction site for the molecule? The molecular-level prediction is probably the standard use case, and so it should be addressed while evaluating site classification models.

### Transformer Models

Molecular transformer models leverage developments in natural language processing which make it possible to translate one language into another.^16^ This technology has been applied to “translating” reactants into products. Molecules can be represented as lines of text using SMILES,^17^ DeepSMILES,^18^ SELFIES,^19^ or other methods, and the relationships between the structures are modeled using the self-attention mechanism which is described in detail elsewhere.^20,21^ Other applications of these models include predicting yields^22^ or reaction class,^23^ keeping track of atoms in a chemical reaction^23^ (atom mapping), and identifying the active sites in enzymes.^24^

**Figure 3:**
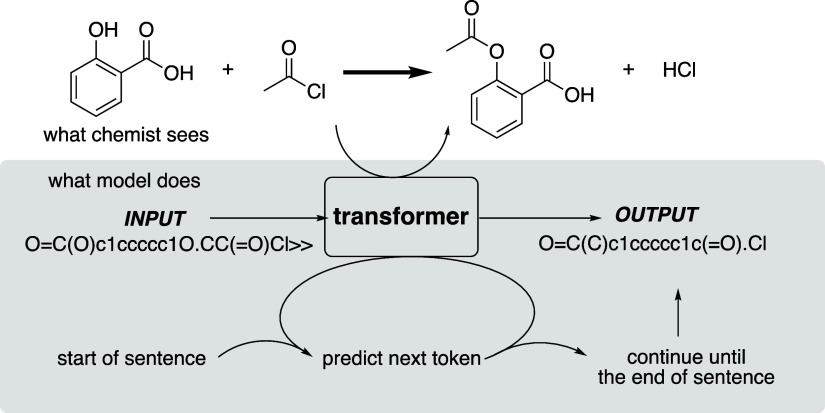
Autoregressive conditional generation of the reaction product by encoder–decoder transformers using character-level tokenization.

Transformers may contain an encoder module, a decoder module, or both. The encoder converts the text input into a context-dependent embedding, i.e., an internal vector representation that takes the relationships between the neighboring tokens into account. The decoder module generates new tokens from this embedding. If the process has been trained on the SMILES representation of a molecule (see Figure 3), then the output should be the SMILES string of a new molecule.

The development of the encoder–decoder transformer architecture^16^ allows us to treat reaction prediction as a translation task, generating products based on reactants and reagents provided. Molecular Transformer^25^ by Schwaller et al. was the first model of this kind to predict reaction products. By reversing the translation direction, the model was successfully repurposed to retrosynthesis tasks.^26^ Subsequent development^27^ improved the prediction quality both for forward and retrosynthesis, enhancing performance for scarce data^28,29^ and increasing the diversity of the possible retrosynthetic disconnections.^30,31^

**Figure 4:**
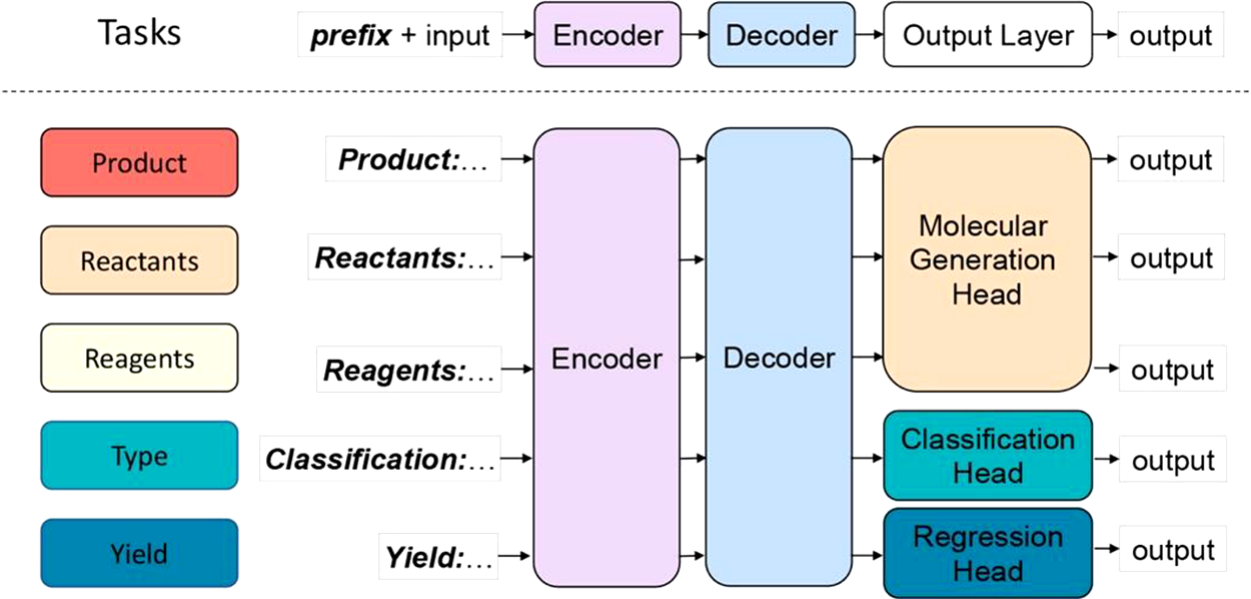
Overview of the T5Chem model architecture. Reprinted with permission from *J. Chem. Inf. Model.***2022**, *62*, 1376–1387. Copyright 2022 American Chemical Society.

For our study, we chose to use T5Chem,^32^ a multitask encoder–decoder model (Figure 4). In addition to SMILES generation as in Molecular Transformer, it can also assign a reaction class and predict a yield using task-specific output layers, known as “heads”, on top of a common encoder–decoder module. In contrast with other encoder–decoder transformers, the authors chose to use primitive character-level tokenization (e.g., “Cl” corresponds to two tokens and “[C@@H]” corresponds to six tokens) rather than the regular expression-based atom-level tokenization proposed by Schwaller.^33^ The reduction in vocabulary size led to a higher prediction accuracy despite the increased number of tokens in a sentence.^28,32^

The T5Chem model is available pretrained on SMILES for molecules encountered in PubChem and tokenized at the character level. This improves the model performance on the downstream prediction tasks despite using less task-specific data, as the model appears to have learned the representation of a molecule. This allows us to use the model for several purposes, thus saving on computational resources.

### Data Curation

**Figure 5:**
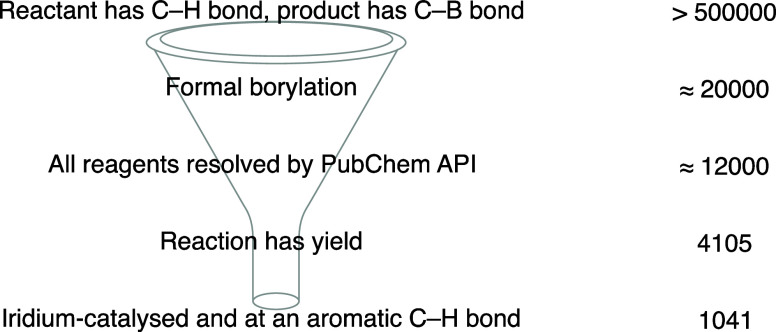
Overview of filtering for the **BORON1000** data set generation.

Data were compiled from Reaxys^34^ and used in this study, as provided by Elsevier Limited under license. A naïve search for a C–H bond in a reactant and a C–B bond in a product resulted in roughly 500 000 transformations. However, many of these are not associated with the C–H borylation of interest (see SI and Figure 1). To further curate our data set, we fragmented the products along the C–B bonds and checked if the fragment structures matched the reactant. That left us with around 20 000 reactions, out of which only about 12,000 had all of the reaction species identified by PubChem. This is necessary, as conversion from the structure to SMILES representation is done by using the PubChem record. Among these, only 4105 had associated yield data and only 1041 involved iridium-catalyzed aromatic borylations. This is comparable to the 1300 reaction set assembled from the literature keyword search of SciFinder reported by Nippa et al.^14^ The resulting set was termed **BORON1000** (Figure 5).

**BORON1000** is limited to aromatic borylations, and while aromatic motifs are common in drugs, further refinement and expansion of the data set are required to capture advances in other borylation classes, including sp^3^-rich substrates.

**Figure 6:**
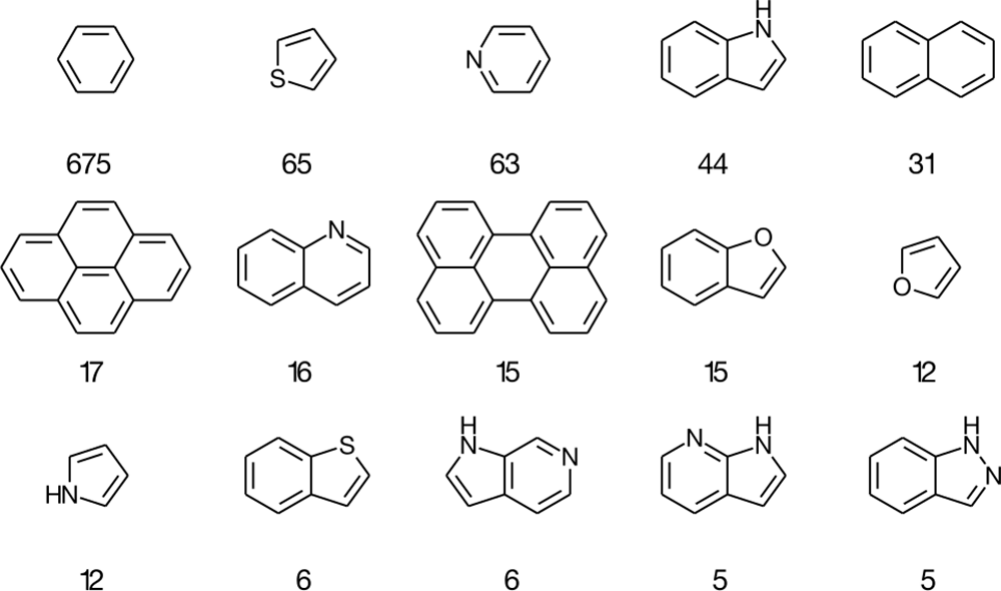
Fifteen of the most common reacting aromatic systems in **BORON1000**.

Enumerating rings in the substrates demonstrates the prevalence of benzenes, with thiophenes, pyridines, indoles, and quinolines also abundant (Figure 6). Such motifs are also found in drug molecules which should make the model applicable to lead development.^35^

### Training Details

The models were trained on a GeForce RTX 3080 instrument for 100 epochs unless specified otherwise. From the original publication, the batch size was reduced from 32 to 16, and the initial learning rate was reduced from 5e-4 to 2.5e-4 accordingly. Character-level tokenization was employed to take advantage of available pretrained models. For molecular generation, the model was set to return five highest probability predictions with a beam search of width ten so that the ten most probable predictions so far are kept during the prediction with the five most probable retained for later.

This study investigates three approaches to the C–H borylation selectivity problem: (A) product SMILES prediction, (B) reaction site classification, and (C) yield prediction. The next three sections of the paper go through these in order.

### (A) C–H Borylation Selectivity Analysis by Product SMILES Prediction

#### Model Development and Evaluation: T5Chem Models for Product SMILES Prediction Task

We set out to study how well the T5Chem^32^ model predicts the borylation products. The metric T5Chem molecular generation employs a top-*k* accuracy which reflects if the first *k* predictions contain a correct answer. In this model, we use RDKit^36^ to turn the SMILES representations of the predicted product into canonical SMILES. For the correct answer, these must be identical to the experimental results. For **BORON1000**, we found that the median number of aromatic C–H bonds is equal to four, so random guessing of the reaction site has a 25% chance of being correct (Table 1).

**Table 1:** Breakdown of **BORON1000** Data by the Type of the Reactive Aromatic System and Number of Aromatic Rings in a Substrate

**BORON1000**	1 aromatic ring	>1 aromatic ring	sum
reactions at carbocycles	530	315	845
reactions at heterocycles	114	82	196
sum	644	397	1041

We took advantage of the existing pretrained models^37^ which were supplied alongside the GitHub repository for the T5Chem model. The first model, **pretrain 1** (denoted as *simple* in the original manuscript), was pretrained on the SMILES representations of molecular structures encountered in PubChem, using masked language modeling.^38^ The model was given SMILES with one character randomly masked and trained to predict the missing token. This helps the model learn the syntax of SMILES.

The **pretrain 1** model was trained further on USPTO_500_MT^32,39^ reaction SMILES data as **pretrain 2** (denoted as *USPTO_500_MT* in the original manuscript) in mixed mode^40^ so that it could perform product, reagents, and reactants prediction.

USPTO_500_MT is a subset of USPTO 1k TPL data set^23^ containing reactions corresponding to 500 most frequent reaction templates and was developed by Lu and Zhang to test how well the T5Chem architecture would handle training for multiple tasks.

**Figure 7:**
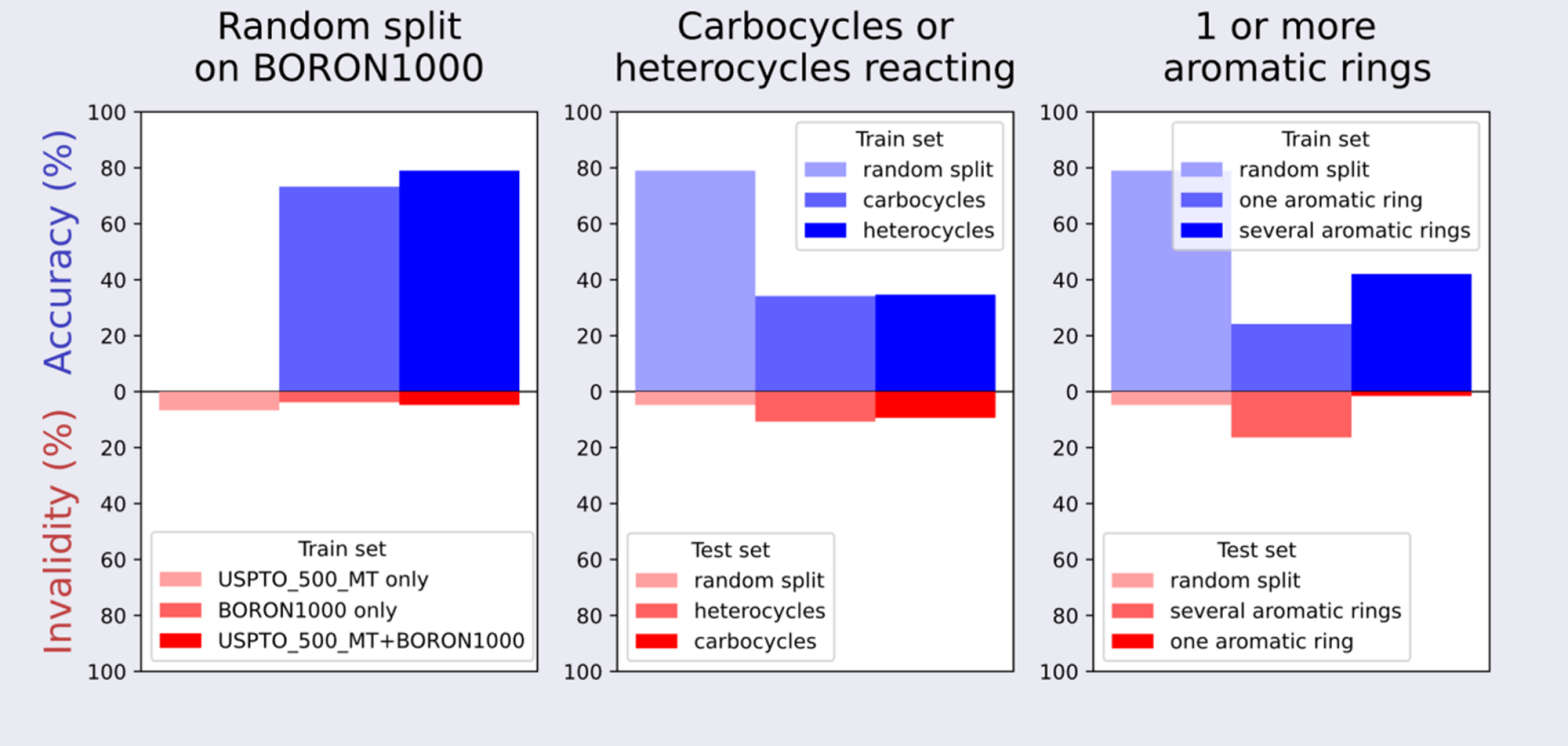
Top-*k* prediction accuracy for the models pretrained from **pretrain 2**.

To establish a baseline, we tested **pretrain 2**, which is pretrained on patent data on the **BORON1000** data set (Figure 7, left). 93% of predictions were syntactically valid, demonstrating that **pretrain 2** has sufficient information about SMILES to generate reasonable molecules. This model was pretrained on the USPTO_500_MT data set which contains no iridium-mediated borylations. As a result, none of the top predictions corresponded to the products in the test set. Encouraged by the level of valid molecules that were generated, we further trained the model by using borylation data.

The **pretrain 1** model was further trained for 100 epochs on borylation data to generate a new model, **finetune 1**. This showed 73% top-1 accuracy (the top molecule was correct), which is a major improvement over the base case. Training the **pretrain 2** model for 100 epochs on borylation data **BORON1000** allowed the model **finetune 2** to generate the correct product structure as the most probable in 79% of cases. The model appears to benefit from further training on translation tasks, as it is getting better conditioned for output generation through exposure to common structural changes in the reactions.

**Figure 8:**
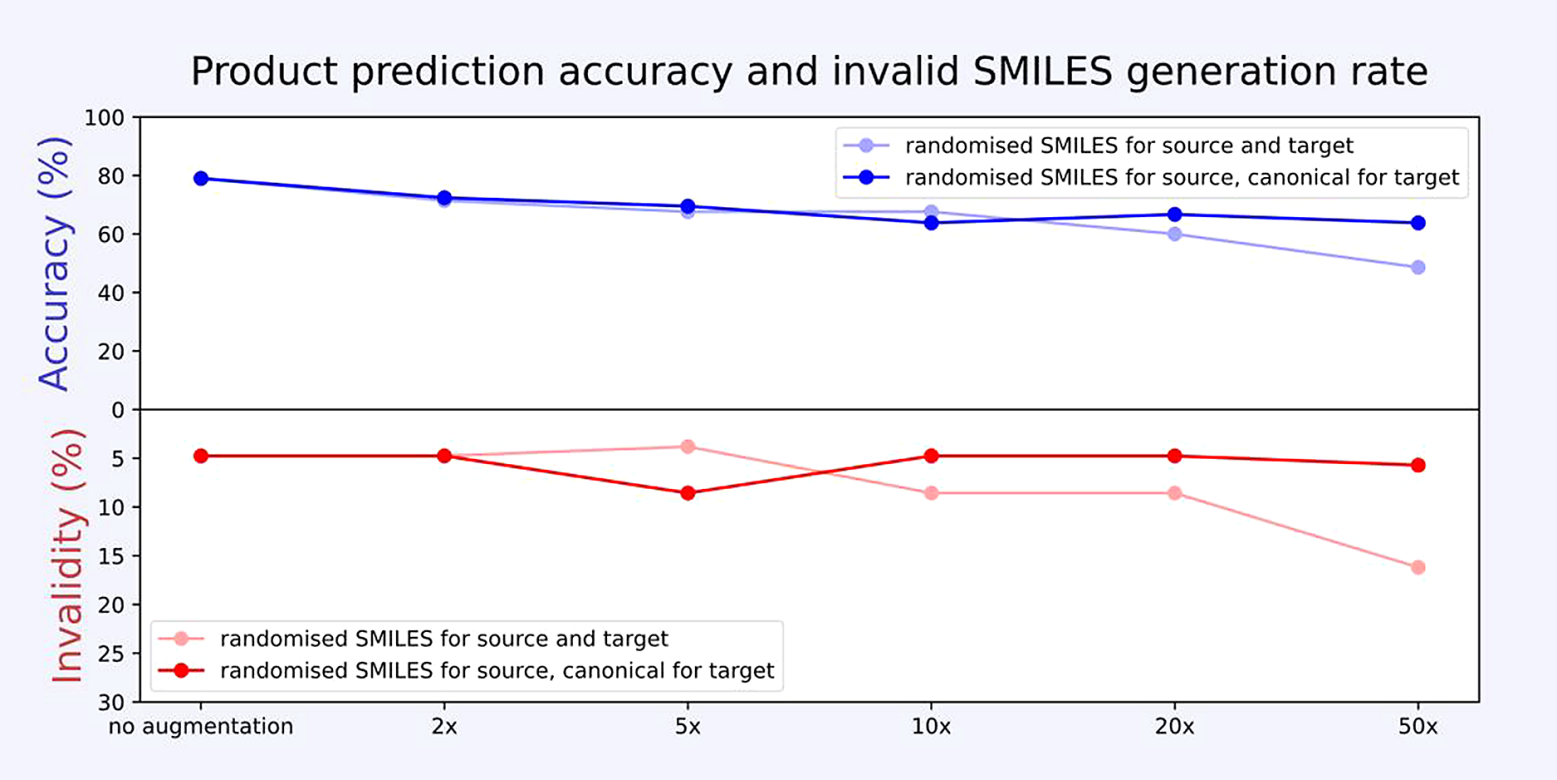
Impact of **BORON1000** data set augmentation on top-1 product prediction accuracy. The accuracy and proportion of syntactically valid SMILES both decrease with the extent of reaction SMILES augmentation in the training set.

Studies into generative language models have shown that training on multiple SMILES representations of the same molecule improves the quality of generated SMILES.^41,42^ However, when trained from **pretrain 2**, we found that the accuracy of borylation product prediction decreases upon augmentation of the **BORON1000** data set (Figure 8), especially when the target SMILES sequence was made noncanonical as well. Therefore, we did not add augmentation to the training data for our models.

#### Scaffold-Based Cross-Validation

In general, random splitting of the data set for model evaluation is not a reliable method for assessing its performance, as it may lead to an overestimation of the model’s accuracy.

To gauge the extrapolative power of the T5Chem model, we split **BORON1000** into sections, as summarized in Table 2. **BORON1000_HET** contains reactions of heterocycles only, and **BORON1000_CARB** contains reactions of carbocycles only. We then trained the **pretrain 2** model on reactions at heterocycles and evaluated on reactions of carbocycles and vice versa to yield **trained_on_heterocycles** and **trained_on_carbocycles**.

**Table 2:** Subsets of **BORON1000** Used in Cross-Validation

subset name	substrate features
**BORON1000_HET**	reacts at a heterocyclic ring
**BORON1000_CARB**	reacts at a carbocyclic ring
**BORON1000_ONE**	has one aromatic ring
**BORON1000_MULT**	has several aromatic rings

The **trained_on_heterocycles** and **trained_on_carbocycles** models still generate syntactically correct SMILES, albeit at a lower rate, but the top-1 accuracy plummets from 79 to 34% for either split (Figure 7, center).

To assess how well the architecture performed in predicting molecules with different levels of structural complexity than those in the training set, we resplit the data set into reactions of molecules with one aromatic ring **BORON1000_ONE** and multiple aromatic rings **BORON1000_MULT**. We then trained the **pretrain 2** model on these sets in the same manner as that above to obtain **trained_on_1** and **trained_on_multiple**.

As the model **trained_on_1** extrapolated to the molecules containing multiple rings, the results showed a 24% top-1 accuracy, with 16% of the predictions being syntactically incorrect. Testing the model trained on polyaromatic molecules **trained_on_multiple** on molecules with one aromatic ring resulted in 42% accuracy with just 1.5% syntactically incorrect predictions (Figure 7, right). All of these new models are listed in Table 3.

**Table 3:** Summary of T5Chem Models Trained for Product SMILES Generation

model	tokenization	trained from	trained on
**pretrain 1**	character-level		PubChem SMILES (masked LM)
**pretrain 2**	character-level	**pretrain 1**	USPTO_500_MT
**finetune 1**	character-level	**pretrain 1**	**BORON1000**, random split
**finetune 2**	character-level	**pretrain 2**	**BORON1000**, random split
**trained_on heterocycles**	character-level	**pretrain 2**	**BORON1000_HET**
**trained_on_carbocycles**	character-level	**pretrain 2**	**BORON1000_CARB**
**trained_on_1**	character-level	**pretrain 2**	**BORON1000_ONE**
**trained_on_multiple**	character-level	**pretrain 2**	**BORON1000_MULT**

#### Comparison of Product Generation by T5Chem Methods and Mechanistic Analysis

**Figure 9:**
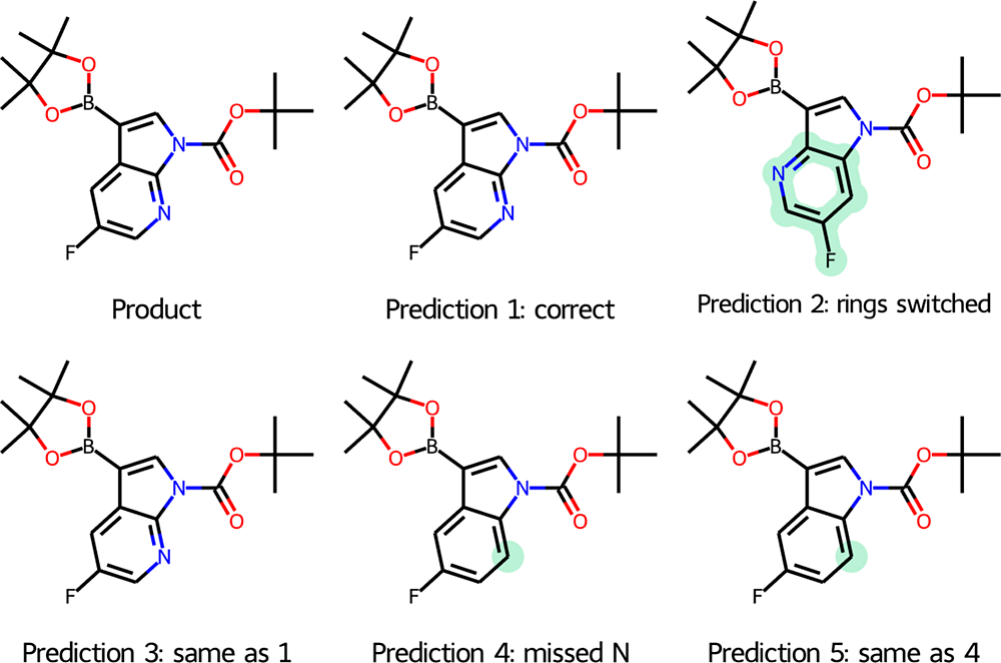
An example of a prediction for a borylation product.

The **finetune 2** model’s lower probability predictions illustrate how the internal representation is gathering the key features of the transformations through the lens of the data that trained it. Changes that look quite dramatic to a chemist with a knowledge of organic synthesis appear to be less significant to the model, as illustrated in Figure 9.

For example, moving a nitrogen within a pyridine ring and changing a pyridine to a carbocycle are difficult synthetically but are small changes in terms of a SMILES string. Branch transposition also appears probable to the model and may well be synthetically challenging. This information, which is “obvious” to humans, is not part of the training set for the model. Fortunately, this extra information can readily be added at the postprocessing stage by checking for substructures in the predicted products. The molecules with major structural changes may be readily filtered out using the starting material as an RDKit substructural filter because any substrate of C–H activation is considered a substructure of a product.

We suggest that reduction in performance for complex structures as shown by the application of model **trained_on_1** to the **BORON1000_MULT** molecules may be a consequence of autoregressive generation, since the probabilities for the next token are dictated by input and output generated so far. For example, a model trained exclusively on structures with one ring, when extrapolating to molecules with multiple rings, having generated one ring, would assign a low probability of generating a token to open another ring, let alone a matching character to close it.

It appears that the model **trained_on_multiple**, that was trained on more complex substrates, can extrapolate to simpler molecules of **BORON1000_ONE** despite fewer training points available. Exposure to complex examples of **BORON1000_MULT** allows for a more robust generation of SMILES strings, but not greater accuracy. This demonstrates the importance of a representative training set as the models did not extrapolate out of training data distribution well.

The unusual negative impact of the augmentation may be caused by the deterioration of the generative capacity of the model. While augmentation by generating multiple distinct SMILES for each molecule can make molecular representation more robust, as the model is exposed to several representations of the same structure, it may also erode the model confidence during generation since during the training, the T5Chem model text generation is evaluated using the cross-entropy loss function. The model loss is minimal if the generated SMILES is identical with the target SMILES, but the generation of different SMILES representing the same molecule is penalized. Further augmentation of the drug concentration increases this problem.

The compiled T5Chem model only supports atom-wise and character-wise SMILES tokenizer. While it would be interesting to explore larger token sizes, which could encode functional groups and other common molecular patterns in a single token,^43^ it was not practical to implement this, as models require complete retraining with each new tokenizer. Atoms with two-letter symbols, such as chlorine, are represented by two tokens rather than by one, which may be counterintuitive. However, in an independent study, the use of data-driven tokenization was shown not to bring about an improvement in molecular generation accuracy.^44^ We decided, therefore, to focus on testing the character-wise tokenizer.

#### Comparing the Generative Model with Quantum Mechanics Calculations

**Figure 10:**
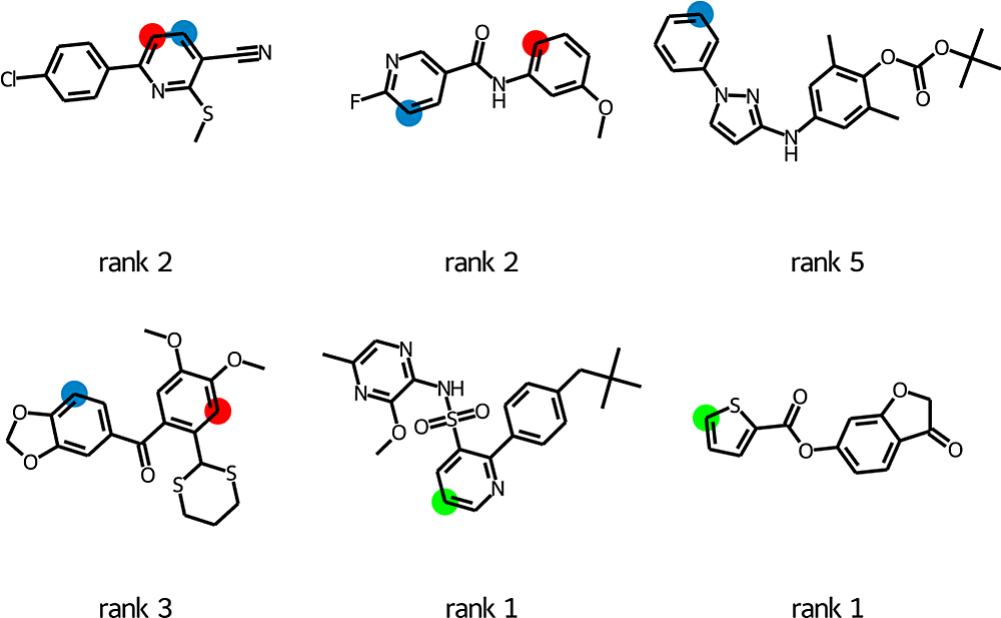
T5Chem is generating predictions for the SoBo validation set of molecules.^13^ The T5Chem predictions are colored red, while the experimental results are shown in blue. The green color indicates a match between the two. For each molecule, the rank of the experimental outcome (ground truth) returned as a prediction by the T5Chem model is also displayed.

For a comparison with the prior art, the model **finetune 2** was tested on the validation set of six pharmaceutical intermediates from the SoBo model paper.^13^ The **finetune 2** model correctly predicted the products for two molecules out of six (Figure 10). For the four erroneous predictions, the model predicted either a wrong site or no reaction. The correct answer was in the top five predictions for all six molecules and in the top two for four out of six. The survey undertaken by the authors of the SoBo paper suggests that our model performs at least on par with an average synthetic chemist. Considering the complexity of the mechanism (Figure 1) and the potential for diverse features of the process to control the outcome, it is remarkable that **finetune 2**, which has no direct knowledge of the mechanism, can be so effective.

### (B) C–H Borylation Selectivity Analysis by Reaction Site Classification

#### Are We Asking the Right Question in the Right Way?

The generative model in the previous section is doing two different things: first, it generates an internal representation of the input molecule in a form which may be suitable for chemistry-related tasks; second, it generates a new molecule based on this information. Even if the first step was performed perfectly, the second step could introduce uncertainty and inaccuracy into the model prediction. If the T5Chem language model has an accurate or reasonably accurate, internal representation of SMILES suitable for chemistry-related tasks, then it should be possible to get useful information from this without going through the process of generating a new molecule as the output. Token classification of molecular SMILES should be able to point out the reactive atoms based on the SMILES of the molecule alone using encoder-only models.^45^ However, the encoder–decoder T5Chem model is typically used to predict a singular output, such as reaction class or yield, and requires adaptation to predict atomic properties such as regioselectivity. Therefore, if we want to predict reacting atoms rather than whole molecules, we must reformulate the question.

#### Site Selectivity via Classification

**Figure 11:**
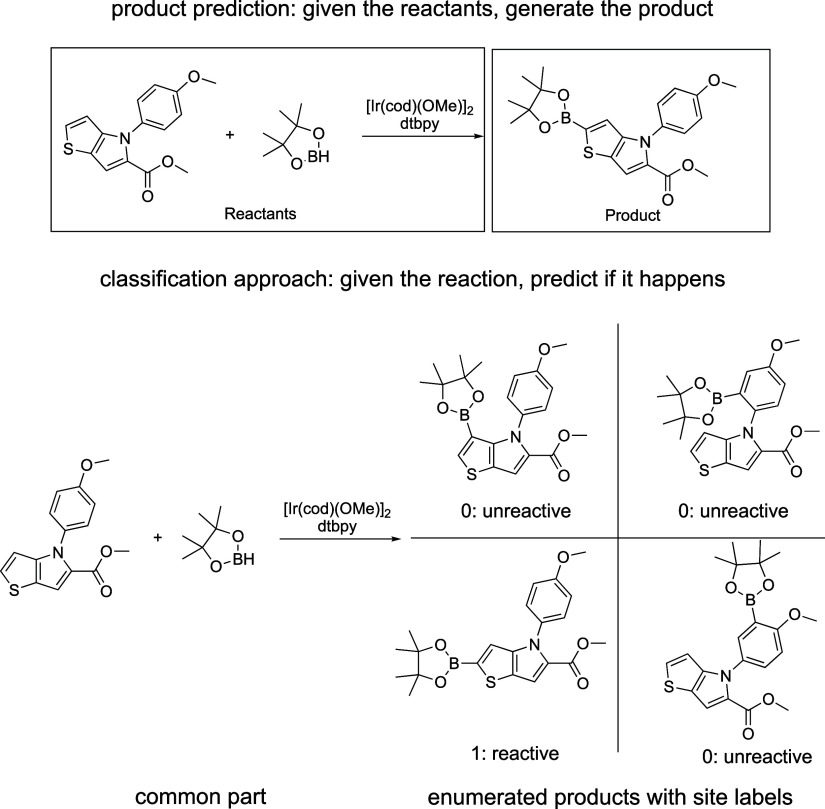
For C–H activation, product prediction may be expressed as a site classification problem. For the T5Chem model, this can be implemented by enumerating all possible monoborylation reactions and determining whether they take place.

We can treat the borylation reaction as an ensemble of reactions for each aromatic C–H bond. All possible monoborylation products can be enumerated and compared with the experimental outcome. Each of these possible reactions are either put into class 1 (reactive) or class 0 (unreactive, Figure 11). This approach has the advantage that negative reactions are specified explicitly, which should improve learning effectiveness, since adversarial examples are now available. In addition, if all sites are classified as nonreactive, we can conclude that the reaction does not happen at all. The output of the model is a list of all the reactive sites of the input molecule.

The classification task shares most of its hidden states with the autoregressive molecular generation, which we have explored previously. However, instead of producing a probability distribution for the next character across the vocabulary space (i.e., “C”, “O”, “1”), it outputs the probability distribution over the two classes (class 0 if the site is not reactive and class 1 otherwise). The model inference runs only once per reaction site, circumventing the demanding task of molecular generation.

Now that the problem has been reduced to binary site classification, it is possible to make meaningful comparisons to other site-classifying models. Due to an imbalance between reactive and unreactive sites, simple accuracy becomes an unreliable metric. We, therefore, choose to use Matthews’ correlation coefficient (MCC)^46,47^ which has been successfully employed as a binary classification metric.

**Figure 12:**
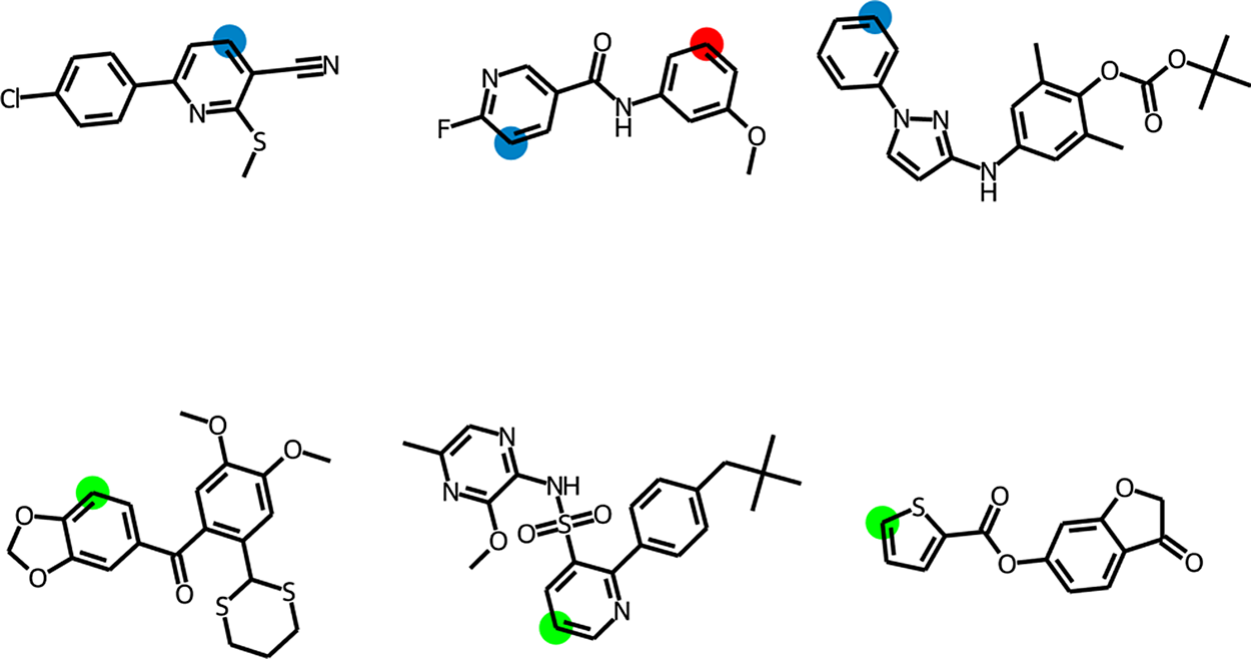
T5Chem Classifier is classifying reaction sites for the SoBo validation set of molecules.^13^ The T5Chem predictions are shown in red, while the experimental results are shown in blue. The green color indicates a match between the two.

We have used the **pretrain 1** model (see Table 3), which was pretrained only on molecular SMILES from PubChem and no reaction data as a baseline. Using the same split as product prediction, we have obtained 95% accuracy in classification, with MCC at 82%. This means that the hidden representation of the molecules in the T5Chem model is sufficient for predicting the reactivity for each aromatic C–H bond in the molecule. Interestingly, using **pretrain 2** as a starting point brought no improvement to the classification accuracy, even though this model had been trained on reactions as well as on molecular structures. We did not try **finetune 1** or **finetune 2** because they had already been trained on the borylation data. Overall, the model predicted the correct selectivity for 84% of **BORON1000** validation set molecules (Figure 12).

**Figure 13:**
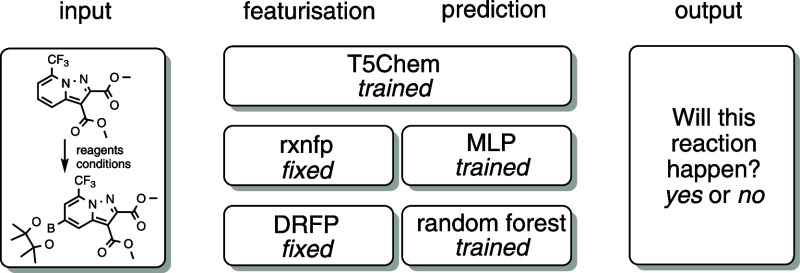
Simpler models were used to estimate the trade-off between the T5Chem classifier complexity and performance.

Transformers are comparatively computationally expensive to train and evaluate. Therefore, a comparison with less sophisticated methods is required to justify their use (Figure 13). For our baseline, we selected the pretrained encoder-only transformer RXNFP^22^ model, which converts reaction SMILES into a feature vector of floating-point numbers and fitted a random forest classifier on top of it to translate this reaction encoding into a reaction site classification. The MCC was 44%, which suggested that further fine-tuning was required. A multilayer perceptron-based classifier improved the MCC to 61%. We also investigated knowledge-agnostic differential reaction fingerprints (DRFPs) which are based on a symmetric set difference of the SMILES representation of molecular features extracted by extended connectivity fingerprints of products and reactants.^48^ The fingerprint is a bit vector (i.e., contains only 0s and 1s) and can be directly matched to the structural features it encodes. We generated 256-bit DRFPs of the same data and fit a random forest classifier using default hyperparameters. We achieved a 93% site classification accuracy with MCC at 79% despite the simplicity of the model. However, the correct reactivity pattern, i.e., all sites in the molecule are classified correctly, was reproduced in only 72% of cases. This demonstrates how a small reduction in the quality of the site classifier may dramatically affect the correctness of the prediction for the entire molecule, highlighting the need for improved accuracy.

**Figure 14:**
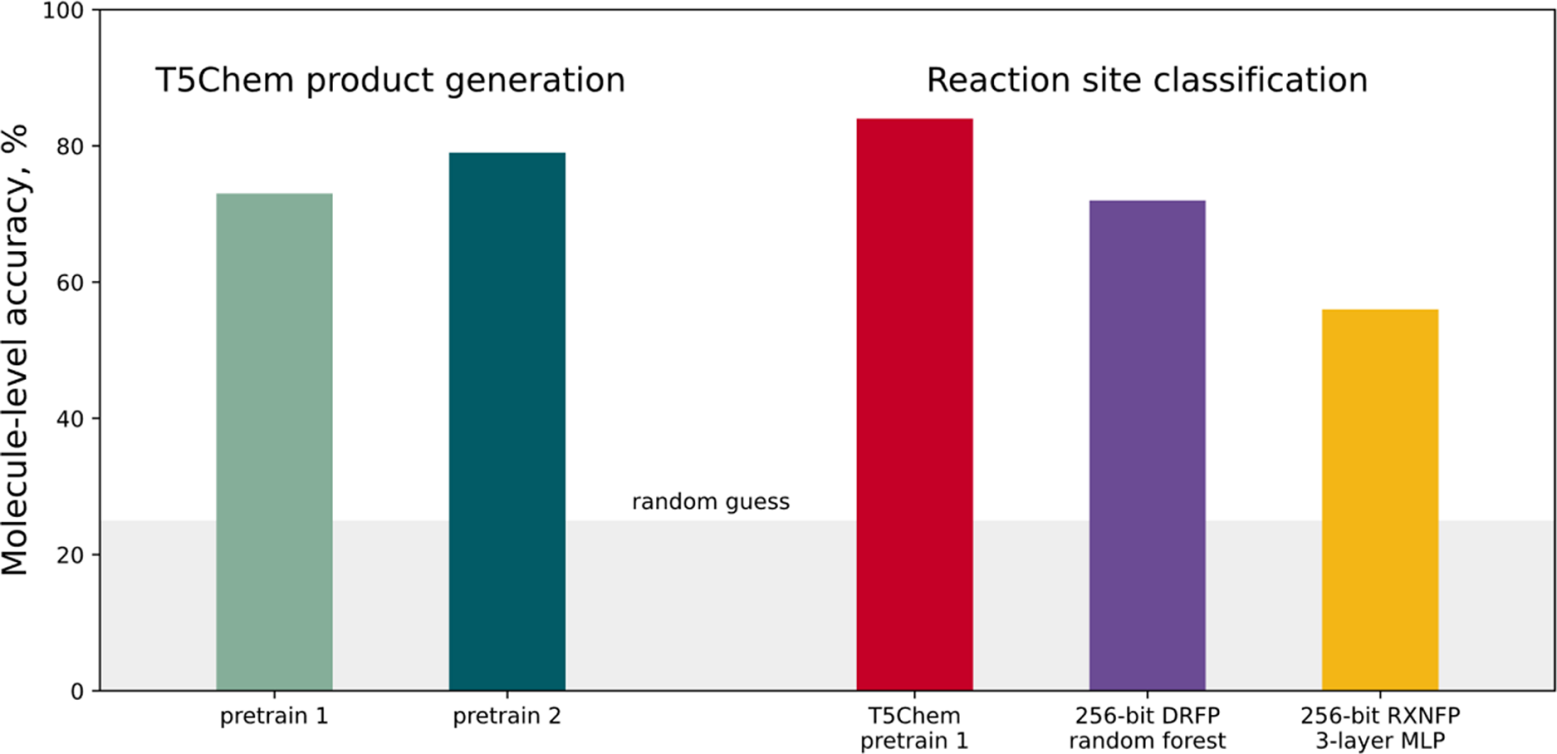
Molecule-level model performance after training and testing on the **BORON1000** data. For the T5Chem product generation task, accuracy is the proportion of correct first predictions; for classification tasks, it requires all sites to be classified correctly.

We expected that the RXNFPs should be able to achieve a better performance because they are more complex than DRFPs. However, the opposite is true. We suspect that the discrepancy is due to the way they were obtained. RXNFPs were trained on a USPTO data set that does not contain iridium-catalyzed borylations, while DRFPs are data agnostic. The embedding generated for an out-of-scope reaction might be of poor quality. On the molecular level, we got a 72% accuracy for the DRFP-based classifier, performing on par with the molecular generation by T5Chem using a simple model with features that directly map onto the structure of a molecule (Figure 14).

**Figure 15:**
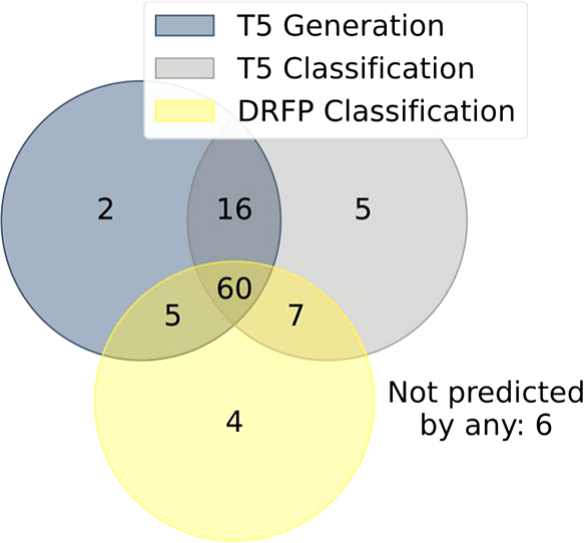
Venn diagram comparing the number of successful selectivity predictions for molecules of various methods on a held-out validation set of **BORON1000**. The set contains 105 molecules in total.

**Figure 16:**
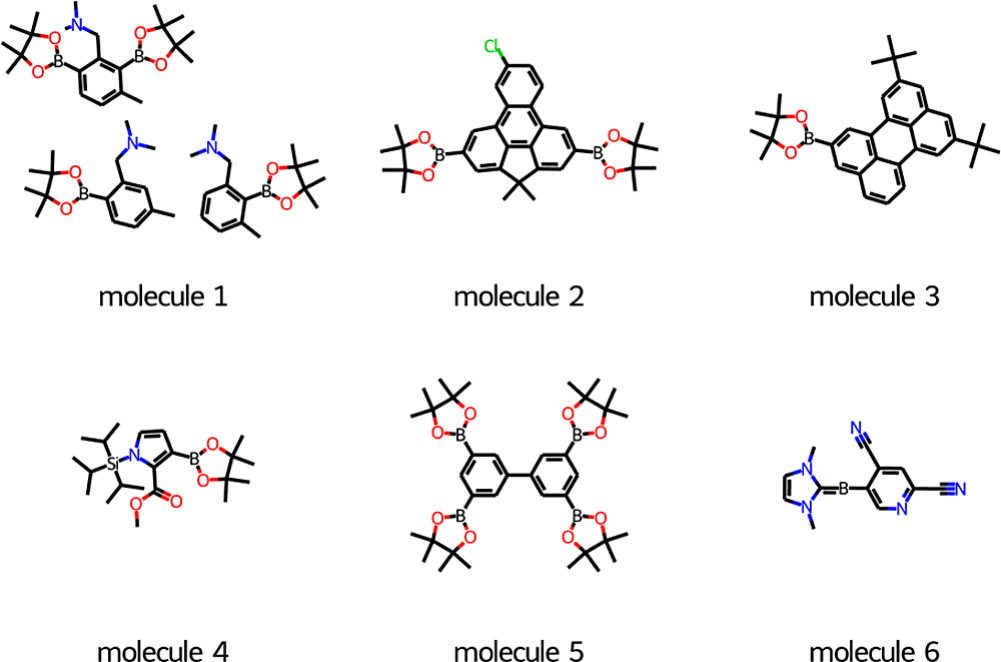
Six hard molecules that T5Chem Generator, T5Chem Classifier, and DRFP Classifier failed to predict correctly.

We compared the DRFP-based model and T5Chem approaches using the same validation data set. We wondered whether the differences in model performance might be due to characteristics of the molecule; i.e., there would be molecules all models would predict correctly and molecules no model would predict successfully. The Venn diagram comparing these predictions (Figure 15) shows there are only six “hard” molecules that no model could predict (Figure 16).

The models appear to struggle with mixtures of products (molecule 1), molecules containing condensed aromatic rings (molecules 2, 3, and 5), or unconventional motifs like *N*-heterocyclic carbene fragment (molecule 6).

**Figure 17:**
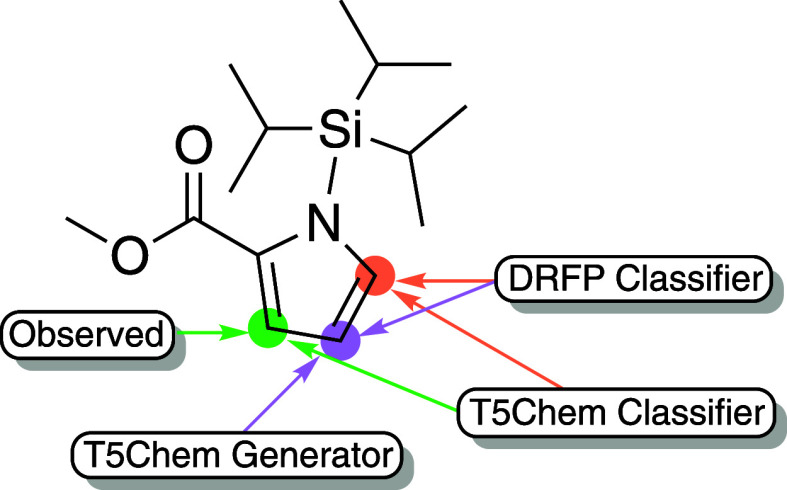
Comparison of the site selectivity prediction using different approaches. The experimental borylation site is highlighted in green.

**Figure 18:**
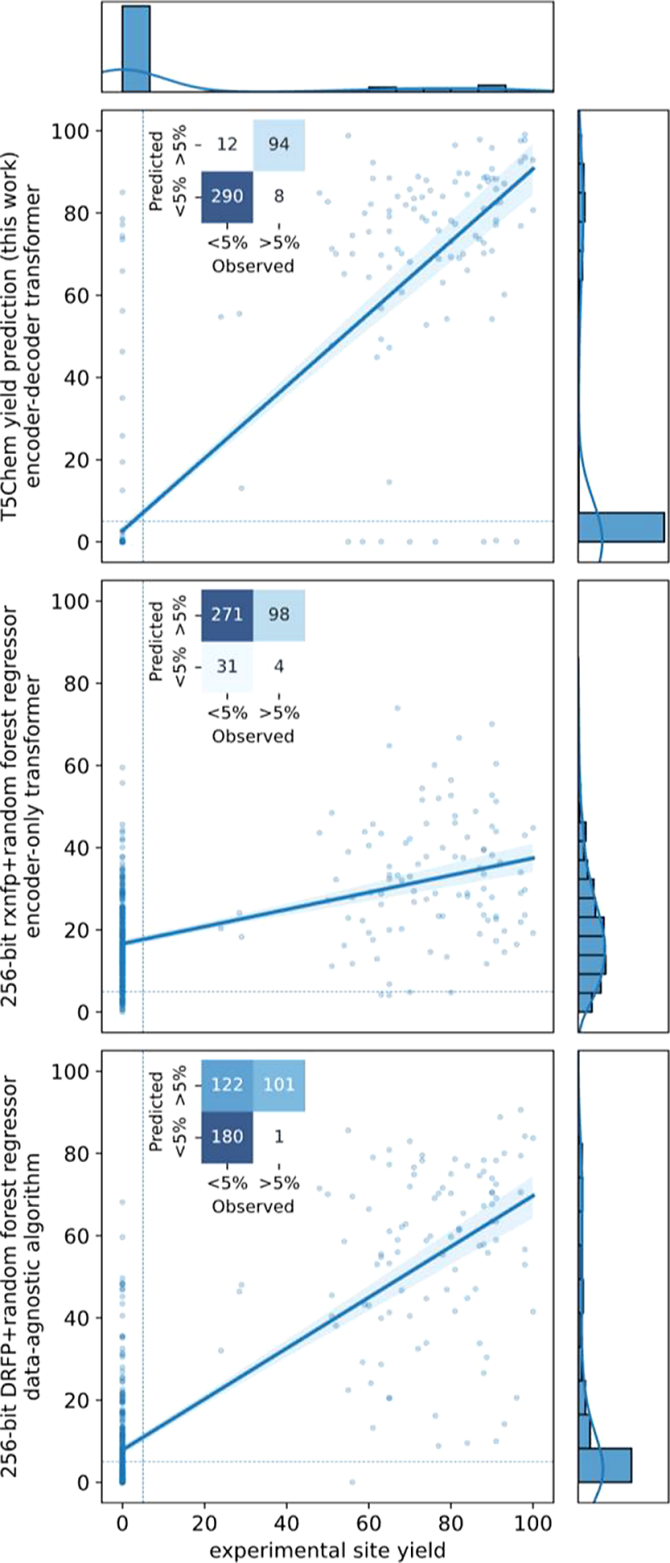
Parity plot for site-level yield predictions by T5Chem (this work), 256-bit RXNFP with random forest regressor, and 256-bit DRFP with random forest regressor as two baseline models. The confusion matrix (top left of each plot) shows the high performance of T5Chem.

While, Figure 18, molecule 4 looks easy to predict using expert-derived rules,^11^ the three models did not arrive at a consensus on which positions would react (Figure 17). While T5Chem Generator proposed a 4-position so that borylation happens at the least hindered site away from both ester and the bulky *N*-triisopropylsilyl protecting group, the actual reaction has taken place at the 3-position, presumably due to ester acting as a Lewis base for iridium. This suggests that the T5Chem Generator may have learned the importance of common steric factors but not chelation.

The classifier models proposed two reactive sites each. The T5Chem Classifier suggested 3 and 5 positions, which match taking chelation and selectivity for 2 and 5 positions of pyrrole. The DRFP Classifier suggested 4 and 5 positions, reflecting the general steric trend and selectivity in pyrroles.

#### Comparison with Existing Site Classification Models

It would be interesting to compare the approach with other site-level regioselectivity predictors, namely, graph neural networks developed by Nippa et al.^14^ We trained the **pretrain 1** model for site classification ten times using randomly split data from their study and found that the site assignment accuracy was 94 ± 1%, positive predictive value (precision) was 84 ± 5%, and F1-score was 82 ± 5%. This is a meaningful improvement over the aGNN3DQM performance reported by Nippa et al.: 90 ± 1%, 62 ± 2%, and 60 ± 4%, respectively. Despite seemingly close accuracy numbers, the multitask T5Chem architecture demonstrates a greater precision than a purpose-built model, enabling greater confidence in its predictions. However, a simple combination of 256-bit DRFP and a random forest achieves the same result with considerably less resource (Table 4).

**Table 4:** Selection of Site Classification Methods Applied to the **BORON1000** Data

	site accuracy/%	PPV/%	MCC/%
T5Chem Classifier	95	87	87
DRFP + RF Classifier	93	94	80
RXNFP + RF Classifier	81	91	44
RXNFP + MLP	85	68	61

### (C) C–H Borylation Selectivity Analysis by Yield Prediction

#### Yield Prediction for Site Selectivity Analysis

We modified our T5Chem classification approach for yield prediction (Figure 18). The *R*^2^ of the regression was 0.75, the mean absolute error was 6, and the root-mean-square error was 17. The error metrics may not be accurate as the data are skewed due to the prevalence of nonreactive sites and misclassified C–H bonds (e.g., yield estimated to be 0% instead of 70%). These may drive a dramatic error increase, especially for the RMSE (Table 5).

**Table 5:** Predictions on the Roche Data Set^a^

	site accuracy/%	PPV/%	F1-score, %
aGNN3DQM^14^	90 ± 1	62 ± 2	60 ± 4
T5Chem Classifier	94 ± 1	84 ± 5	79 ± 5
DRFP + RF Classifier	94 ± 1	95 ± 3	80 ± 3

a For consistency, the models were trained on a data set prepared by Roche to classify all nonquaternary carbons, average of 10 random splits listed.

Using Kullback–Leibler divergence as the loss function, the T5Chem-based yield prediction reproduced the distribution of yields in the test set (Figure 18). To compare the model with the baselines, we used the same sets of RXNFPs and DRFPs and fit a random forest regressor on top of those. We found that the RXNFP-based model does not reproduce the distribution well with *R*^2^ at 0.25. The DRFP-based model achieved an *R*^2^ of 0.67, outperforming an RXNFP-based model and reproducing the target distribution more accurately.

We set a classification threshold at the 5% yield and obtained an MCC value of 87% which is comparable to that of the classification approach. The consistent performance is reasonable to expect since the model shares all hidden states, except the task-specific heads, which both constitute a linear transform of the same feature vector (see Figure 4). Aggregating those predictions by molecule showed 85% accuracy for a major product.

The classification and yield prediction heads both comprise a linear transform and an output layer but produce the final output differently. The classification head assigns an arbitrary score to each class (in this case, class 0 if there is no reaction and class 1 otherwise) and returns the class with a higher score. The yield prediction head uses a soft label approach so that the outputs correspond to the probability distribution between the minimum label (0) and maximum (100) and are then normalized to produce the yield. We hope this will prevent model overconfidence by forcing it to consider the likelihood of an alternative outcome, as well (Table 6).

**Table 6:** Yield Prediction Accuracy Was Assessed by Site-Level Regressor Models

model	*R*^2^	reaction outcome, MCC	molecule-level accuracy, %
T5Chem	0.75	0.87	85
RXNFP + random forest	0.25	0.10	2
DRFP + random forest	0.67	0.51	39

While this approach can quantify the regioselectivity, we may be limited by reporting bias as reactions resulting in mixtures may not have all of their products listed and yields omitted, as the lack of reactions with yields under 50% would suggest. Having a representative set of reactions would hopefully improve the model performance on novel molecules.

## Conclusions

Using a model based on T5Chem, we can treat reaction selectivity prediction within the same architecture in three distinct ways: generation of a product SMILES, reaction site classification, and site-wise reaction yield prediction.

SMILES generation is the most challenging because molecules are generated from scratch and there is the potential to generate products that are completely unlike the starting materials. While some distant products are generated, sensible products that show the expected reactivity are generated in 79% of cases. When tested on a validation set for the SoBo^13^ model, it predicted selectivity for two out of six substrates correctly on par with a synthetic chemist with no expertise in borylation. Reaction site classification with T5Chem is a more straightforward task because it selects between the possible reactive sites of the starting material rather than generates a completely new molecule. These restrictions lead to it being more effective with 84% molecular-level accuracy at the cost of universal reaction applicability. The model also performed better on the SoBo validation set, predicting selectivity correctly for 3 molecules out of 6, putting it above an average synthetic chemist. Predicting selectivity from T5Chem yield calculations also fits the data well with an *R*^2^ score of 0.75 and can predict the reaction success (yield ≥ 5%) on par with the T5Chem classifier.

The best model for predicting C–H borylation selectivity is the T5Chem site classification model. This works in the absence of detailed knowledge of the reaction mechanism (Figure 1). The model can be readily configured for use by people having no computational experience and trained in one command. Another advantage of fine-tuning the existing model is low resource demand: it only takes about 20 min of consumer-grade GPU time to train the model on 1000 reactions, with predictions returned in seconds. When trained on the same data set, the T5Chem classifier outperforms existing purpose-built graph neural networks,^14^ with a higher *F*-score (78% vs 55%) despite encoding no steric and electronic information about the substrate. Moreover, unlike quantum mechanics-based methods,^13^ we are not restricted to a single reacting system and a singular reaction mechanism, allowing for greater flexibility in applying the model across a broad range of chemical systems and in a mechanism-agnostic fashion.

Overall, we believe it is now possible to predict selectivity for a complex reaction well enough to be helpful to many synthetic chemists, without any mechanistic knowledge or need for purpose-built models.

## Data Availability

A GitHub repository with the scripts employed for data processing is available at https://github.com/ruslankotl/rxn-data-proc. The repository also contains borylation regioselectivity data set as prepared by Nippa et al.^14^ and Reaxys IDs for the reactions that went into **BORON1000** data set. Code to run the T5Chem model is available at https://github.com/HelloJocelynLu/t5chem/tree/main.

## Supporting Information Available

Final MLP classifier hyperparameters, hyperparameters varied in the cross-validation for MLP classifiers, and performance of classification methods as applied to **BORON1000** data (PDF)
