# Supplementary material for: Leveraging Language Model Multitasking To Predict C–H Borylation Selectivity
Source: J Chem Inf Model. 2024 May 6;64(10):4286–97. doi: 10.1021/acs.jcim.4c00137 (PMC11134489; doi:10.1021/acs.jcim.4c00137)
Supplement: Supplementary file 1 — ci4c00137_si_001.pdf [file ci4c00137_si_001.pdf]

Supplementary Information: Leveraging Language Model Multi-Tasking to Predict C–H Borylation Selectivity

Ruslan Kotlyarov[1], Konstantinos Papachristos[2], Geoffrey P. F. Wood[2], Jonathan M. Goodman[1]*

1 Yusuf Hamied Department of Chemistry, Lensfield Road, Cambridge CB2 1EW

2 Exscientia plc, The Schrödinger Building Oxford Science Park, Oxford OX4 4GE

* Email: jmg11@cam.ac.uk

1. Site classification methods

To compare T5Chem classification to simpler models, we have generated 256-long feature vectors from reaction SMILES using DRFP and RXNFP. These have the identical shape to the last hidden state of the T5Chem model and should help us determine if its performance justifies the cost of training and inference (see Table 3).

The data was split in the same way as for the T5Chem model. RXNFPs were either provided as is (unscaled) or scaled to zero mean and unit variance (scaled). Scikit-learn version 1.1.0 was used to compute the metrics.

1.1 Multilayer perceptrons

The dimensions of input and output layers were fixed at 256 and 1, respectively. For the multi-layer perceptrons (MLPs), we have determined optimal hyperparameters (listed in Table 1) by the grid-search cross-validation (Table 2), using ADAM as optimizer, and Matthews' correlation coefficient as the final comparison metric.

Table 1. Final MLP Classifier hyperparameters.

RXNFP	Learning rate	Alpha regularization	Layer dimensions
unscaled	0.001	0.000001	256, 16, 4, 1
scaled	0.001	0.01	256, 16, 1

Table 2. Hyperparameters varied in the cross-validation for MLP classifiers.

Variable hyperparameter	values
Learning rates	0.1, 0.01, 0.001, 0.0001, 0.00001
Alpha regularization term	0.1, 0.01, 0.001, 0.0001, 0.00001, 0.000001
Hidden layer sizes	(2), (4), (16), (16,4), (16,2)

1.2 Random forest (RF)

To account for imbalance between reactive and unreactive site, we tested if applying weights scaling (essentially dividing weights by class prevalence) impacts classification accuracy. No notable performance gain was obtained, and default hyperparameters were used in subsequent experiments.

1.3 Support vector machine

Support vector classifier (SVC) was used with as implemented with default hyperparameters.

1.4 K-Nearest Neighbors

K-neighbors classifier was used as is (KNN) or with Neighborhood Component Analysis as a prior dimensionality reduction technique (NCA+KNN).

Table 3. Performance of classification methods as applied to BORON1000 data.

method	Accuracy, %	PPV, %	MCC, %
RXNFP+MLP, unscaled	82	67	50
RXNFP+MLP, scaled	85	68	61
RXNFP+RF, unscaled	81	91	44
RXNFP+RF, scaled	81	90	43
RXNFP+RF, unscaled and balanced	80	89	40
DRFP+RF	93	94	80
DRFP+RF, balanced	93	96	82
RXNFP+SVC, unscaled	75	0	0
RXNFP+SVC, scaled	77	85	25
DRFP+SVC	89	92	71
RXNFP+KNN, unscaled	79	61	38
DRFP+KNN	89	75	73
RXNFP+NCA+KNN, unscaled	80	66	44
DRFP+NCA+KNN	94	91	83
